# Determinants of Dyadic Relationship and Its Psychosocial Impact in Patients with Parkinson's Disease and Their Spouses

**DOI:** 10.1155/2017/4697052

**Published:** 2017-02-14

**Authors:** Michaela Karlstedt, Seyed-Mohammad Fereshtehnejad, Dag Aarsland, Johan Lökk

**Affiliations:** ^1^Karolinska Institutet, Department of Neurobiology, Care Sciences and Society, Division of Clinical Geriatrics, Novum Pl 5, Blickagången 6/Hälsovägen 7, 14157 Huddinge, Sweden; ^2^Department of Neurology and Neurosurgery, McGill University, Montreal, QC, Canada; ^3^Karolinska Institutet, Department of Neurobiology, Care Sciences and Society, Division of Neurogeriatrics, Novum Pl 5, Blickagången 6/Hälsovägen 7, 14157 Huddinge, Sweden

## Abstract

The caregiver-care receiver relationship (mutuality) in Parkinson's disease (PD) and its association with motor and non-motors symptoms, health-related quality of life (HRQoL), and caregiver burden have not fully been investigated. The aim of our study was to explore if (1) the level of mutuality perceived by PD-patients and PD-partners differs, (2) different factors are associated with perceived mutuality by PD-patients and PD-partners, and (3) mutuality is associated with PD-patients health-related quality of life (HRQoL) and caregiver burden. We collected data on motor signs (UPDRS III), non-motor manifestations (NMSQuest), PD-patients' cognition (IQCODE), mutuality scale (MS), PD-patients' HRQoL (PDQ8), and caregiver burden (CB) from 51 PD dyads. Predictors were identified using multivariate regression analyses. Overall, the dyads rated their own mutuality as high with no significant difference between the dyads except for the dimension of reciprocity. PD-patients' MS score (*p* = .001) and NMSQuest (*p* ≤ .001) were significant predictors of PDQ8. Strongest predictor of CB was PD-partners' MS score (<.001) and IQCODE (*p* = .050). In general, it seems that non-motor symptoms contribute to a larger extent to the mutual relationship in PD-affected dyads than motor disabilities.

## 1. Introduction

Parkinson's disease (PD) is a complex neurodegenerative disorder resulting in a combination of motor impairment and a wide range of non-motor manifestations. Non-motor symptoms (NMS) can emerge from nearly all organ systems, such as neuropsychiatric, gastrointestinal, urogenital, and other autonomic presentations partly due to extrastriatal brain changes [1].

A body of evidence suggests that PD as a progressive disabling condition may lead to not only lack of autonomy due to increasing dependency but also placing an increased burden on caregivers and consequently has an impact on the care dyads' health-related quality of life (HRQoL) [1–5]. The construct of HRQoL is complex but can be defined as the “perception and self-evaluation regarding the impact of the disease and its consequences on his/her life in the terms of physical, psychological and social aspects” [4, 6].

Not all family members may regard themselves as caregivers, especially when symptoms are less severe in the early stage of PD. However, the inevitable course of the disease may result in functional dependency and need of help in order to perform daily activities. This can transform the quotidian caregiving activities and lead to emotional, social, and economic strain [7, 8].

An important aspect of caregiving situation is the relationship between the caregiver and care receiver. Research has demonstrated that the quality of relationship can affect caregiver outcomes [9, 10]. Mutuality, defined by Archbold and colleges as the positive quality of relationship, is now widely used to signify relationship quality [10–12]. Mutuality has four dimensions: love and affection, shared pleasurable activities, shared values, and reciprocity [11, 12]. A review suggests that high mutuality can be an important protective factor against caregiver burden in progressive conditions such as PD [10]. Conversely, low mutuality can be a risk factor for increased caregiving burden and depression for the caregivers. So far, the effect of mutuality has mainly been explored in PD caregiver samples [9, 13, 14]. Very few studies have explored mutuality from the perspective of the PD-patients and their partners and these studies have mainly been based on small sample sizes. Nonetheless, their result has suggested that perceived dyadic benefits of living with PD are associated with greater marital quality and that mutuality may act as mediator of PD-patients HRQoL [15, 16]. In contrast to studies with frail elderly and stroke patients [17, 18] Ricciardi et al. found PD-patients to be less satisfied with relationship than their partner. Furthermore, they did not find any association between mutuality and PD motor impairment or disease duration [19]. More research is needed particularly on the relationship of mutuality and motor and NMS.

Therefore, the aim of this study was to identify factors associated with mutuality, HRQoL, and caregiver burden. We used the modified stress-appraisal model proposed by Greenwell et al. (2015) to guide us in our analytic plan [9, 20]. The model suggests that primary stressors (e.g., disease-related factors) and the individuals' appraisal of the situation (e.g., carer involvement, coping strategies) have direct and indirect effects via protective factors such as mutuality on caregiver burden and HRQoL. Mutuality is also proposed to have a direct effect on caregiver burden and HRQoL. In the present study we only explored primary stressors association with mutuality and outcomes such as PD-patients HRQoL and caregiver burden. We hypothesized that there are (1) differences of perceived level of mutuality by PD-patients and PD-partners, (2) differences in factors associated with the mutuality of PD-patients and PD-partners, and (3) a relationship between mutuality perceived by PD-patients and PD-partners as well as PD-patients' HRQoL and caregiver burden.

## 2. Materials and Method

### 2.1. Participants

In the present study, we report results from baseline data of a longitudinal study. Fifty-one PD dyads were recruited during 2014-2015, from the movement disorders clinics at Karolinska University Hospital (*n* = 42), Sweden, and through advertisement in the journal of the Swedish Parkinson's Disease Association (*n* = 9). The study was approved by the local research ethics committee (registration number: 2013/1812-31/3) and was conducted in accordance with the declaration of Helsinki.

### 2.2. Eligibility Criteria

To be included in the study, a specialist in movement disorders should have diagnosed the PD-patient. They should be living together as partners (≥3 years), aged ≥55, but should not be in the phase of parenting small children. Furthermore, none of the PD-partners should be employed as a caregiver. Other eligibility criteria consisted of acceptable cognition based on Montreal Cognitive Assessment (MoCA, [score ≥ 23]) and no severe medical conditions other than PD affecting daily life, which was judged by MK.

### 2.3. Procedure

The clinical examinations were performed by MK. The care dyads filled out the questionnaires' separately and individually, in the presence of the first author, at the outpatient clinic or during a home visit whichever was most convenient for the dyads. The questionnaires were filled out after having obtained, read, and signed a written consent. Descriptive and sociodemographic data was also collected.

### 2.4. Measurements

#### 2.4.1. Dependent Variables

PD-specific HRQoL was measured with the Parkinson's Disease Questionnaire-Short Form (PDQ8). The scale comprises 8 items, using 5-point Likert scale, and covers domains as mobility, activities of daily life, emotional wellbeing, stigma, social support, cognition, communication, and bodily discomfort. A summary index (PDQ8SI) was calculated as the sum of items divided by maximum per item times number of items and then multiplied by 100. Higher scores, ranging from 0 to 100% indicate worse quality of life [21].

The caregiver's burden scale (CBS) was used to measure the PD-partners' reaction to caregiving. The scale contains 22 items and is answered using a 4-point Likert scale (1 = not at all to 4 = often). It covers domains such as general strain, isolation, disappointment, and emotional involvement. The total scale score ranges from 22 to 88. Higher score indicates more feelings of stress and burden in the caregiving situation [22]. The CBS has been used in samples of patients with Parkinson's disease and other neurological disorders [22–24].

#### 2.4.2. Dependent and Predictor Variable

The quality of the caregiver-care receiver relationship was measured through the mutuality scale (MS) [11, 12]. The scale contains 15 items, where each item is answered using a 5-point Likert scale (0 = not at all to 4 = a great deal). It covers domains such as love and affection (3 items), shared pleasurable activates (4 items), shared values (2 items), and reciprocity (6 items) [12, 18]. The summary score is calculated as the mean value of all the individual items' scores for the whole scale and the above-mentioned domains. The total scale score ranges from 0 to 4. Higher scores indicate better quality of mutual relationship between the care dyads [11, 12]. We have recently reported the psychometric properties of the Swedish version of MS [25].

#### 2.4.3. Predictor Variables

We used the Hoehn and Yahr (H/Y) scale to determine stage of PD. It contains 6 stages where 0 indicates no visible symptoms and 5 represents a PD-patient who is unable to walk unless assisted [26].

The Unified Parkinson's Disease Rating Scale-Part III (UPDRS III) was used to evaluate severity of PD-specific motor signs. The scale contains 14 items and is answered using a 5-point Likert scale. Higher scores indicate more severe motor signs [27].

The Non-motor Symptom Questionnaire (NMSQuest) was used to detect PD-specific non-motor manifestations in domains such as gastrointestinal, urinary, sexual function, cardiovascular, attention/memory, hallucination, depression/anxiety, sleep/fatigue, and miscellaneous. The scale contains 30 items scored “yes” or “no.” Higher score indicates higher frequency of non-motor manifestations [28].

Informant Questionnaire on Cognitive Decline in the Elderly (IQCODE) uses information from the caregiver to assess functional changes associated with cognitive functioning in the patients under care. The scale contains 26 items and is answered using a 5-point Likert scale. The individual score is calculated by the mean across all item scores, ranging between 1 and 5. Higher score (>3) indicates decline in cognitive functioning [29].

The PD-patients' physical functioning and level of dependency were assessed by the PD-partner using the modified form of the extended Katz index [30]. The scale comprises items assessing grooming/dressing, bathing, food intake, toileting, walking/transferring, housekeeping, and shopping. The scale is answered using a 4-point Likert scale (0 = no help to 3 = need all help). A dichotomous variable (0 = independent and 1 = dependent) was created aiming to assess dependency.

We also created a pooled dichotomous variable of the level of education of the PD-patient and the PD-partner (0 = either elementary, secondary, or only one with university education, 1 = both with university education).

#### 2.4.4. Montreal Cognitive Assessment (MoCA)

To assess cognitive functioning, the MoCA screening instrument was used. Scores above 26 are considered to be normal [31].

### 2.5. Statistical Analysis

Continuous and discrete numerical variables are described using mean and standard deviation (SD), whereas stages of PD assessed by H/Y are presented as the median and interquartile range (IQR). Nominal and categorical data are reported as relative frequency and percentages. Prior to the main analyses, we explored the normality of the distribution of all the dependent variables (DVs). Most of the dependent variables' total score were normally distributed with no excessive skewness or kurtosis. Spearman correlation coefficient was calculated to assess direction and magnitude of the correlation between potential predictors and the DVs. Correlation coefficients between 0.1 and 0.29 were considered as weak, 0.3 and 0.49 as medium, and >0.5 as strong [32]. Predictors with correlation coefficients > 0.1 were entered into the multivariate regression models. Our a priori hypotheses on the relationship between the included variables were guided by the aim of the study and the stress-appraisal model [20]. This means that disease-related factors, PD-patients, and PD-partners mutuality may be potential predictors of PD-patients' HRQoL and caregiver burden. Furthermore, disease-related factors may also predict mutuality.

Separate regression analyses were performed for each group and for each dependent variable; that is, we performed one regression analysis including predictors of PD-patients' MS and one including predictors of PD-patients' HRQoL. Furthermore, one regression analysis was performed with predictors of PD-partners' MS and one including predictors of caregiver burden. PD-partners' gender, age, and education were used for statistical adjustments. Assumptions of linearity, normality, and homoscedasticity were examined through histogram and scatterplots of residuals (Table 5). No influential multivariate outliers were detected using Mahalanobis and Cook's distance (<1) [33]. The Mann–Whitney *U* test was used to test the differences of MS total scores and the dimension scores between PD-patients and PD-partners. Prior to data collection, sample size was calculated based on available data from previous studies reporting differences in MS scores between caregivers and care receivers [13, 17]. To detect a standardized difference of 0.63 between PD-patients and PD-partners, with a power of 80% and a two-sided significance level of 0.05, a total of 40 subjects in each group is required. To take into account possible drop-outs due to the longitudinal design of the project, 51 dyads were recruited. All data analyses were conducted using IBM SPSS Statistics for Windows, version 23 (IBM Corp., Armonk, NY, USA).

## 3. Results

### 3.1. Missing Data

Two single missing items by two subjects within the NMSQuest scale were identified. These study subjects had individual scores larger than the samples median. To avoid case-wise deletion and loss of power, these missing values were imputed with a zero score.

### 3.2. Baseline Characteristics

Mean age for the PD-patients was 70.9 (SD = 8.5) and 70.7 (SD = 9.3) years for the partners. Mean length of cohabitation was 38.4 (SD = 14.5) years. Other sociodemographic and clinical features are presented in Table 1. The most frequent reported NMS was nocturia (78.4%) and urgency (74.5%) (Table 2). Of the PD-patients 35/51 (68.6%) needed some form of supervision or help in daily activities. When help was needed the PD-partner was the main provider of that help. Instrumental activities such as shopping (32/51) or cooking/cleaning (28/51) were the most frequent tasks requiring help from the PD-partners (Table 3). Two PD-patients out of 51 (4%) were unable to be left alone in the home and 33% (17/51) could be alone between 2 and 12 hours. The remaining 63% (32/51) were able to be alone unlimited time.

### 3.3. Dyadic Differences in Total MS Score and Dimension Score

There was no significant difference between the total scores of the MS in PD-patients (median = 3.4) and PD-partners (median = 3.1). Regarding dimensions of the MS, only reciprocity (median = 3.3 versus median = 2.8, *p* = .014) was significantly higher rated by PD-patients (Table 1).

### 3.4. Bivariate Correlations

Table 4 summaries bivariate correlation coefficients between predictors and dependent variables. There was a significant correlation between PD-partners MS score and PD-patients MS score (rho = .524, *p* ≤ .001). PD-patients' MS score had a significantly inverse correlation with PDQ8S (rho = −.516, *p* ≤ .001) and UPDRS III (rho = −.311, *p* = .026) but not with caregiver burden and NMSQuest. PD-partners' MS score showed a significant inverse correlation with caregiver burden (rho = −.631, *p* ≤ .001), PDQ8SI (rho = −.409, *p* = .003), and IQCODE (rho = −.529, *p* ≤ .001) but not with NMSQuest and UPDRS III. Hoehn and Yahr stages had a significant correlation with mutuality, PD-patients' HRQoL, and caregiver burden.

### 3.5. Multivariate Linear Regression Analysis

Suspect multicollinearity (tolerance = ≤ .5, rho = ≥ .5) was detected between some of the included predictors. They were removed one by one and the variable that remained was the one with tolerance >.5, highest adjusted *R*^2^ value, and the best fit regarding the assumptions of regression analysis. Contribution of each predictor to explain variance in the final multivariate regression models is presented in Table 5.

#### 3.5.1. PD-Patients' Mutuality

In the final model with PD-patients' MS as the DV, the included predictors explained 31.6% of the variance. Of them, PD-partners' MS score (beta = .419, *p* = .002) and gender of the PD-partners (beta = .332, *p* = .017) contributed most of the explained variance. Consequently, PD-patients' mutuality score was higher in those with a male partner and partners with high level of mutuality.

#### 3.5.2. PD-Patients' HRQoL

With PDQ8SI as the DV, the included predictors explained 49.7% of the variance. PD-patients' MS score (beta = −.433, *p* = .001) and NMSQuest score (beta = .498,* p* ≤ .001) contributed significantly to the explained variance of PDQ8SI scores. In other words, patients with high level of mutuality had significantly better HRQoL (lower PDQ8SI), while an increasing frequency of NMS decreases the HRQoL.

#### 3.5.3. PD-Partners' Mutuality

The included predictors explained 28.9% of the variability in PD-partners' MS scores. PD-patients' MS score (beta = .461, *p* = .002) and increased impairment of cognition (beta = −.314, *p* = 0.016) contributed significantly to PD-partners' mutuality.

#### 3.5.4. Caregiver Burden

The explained variance of the included predictors in the model with CBS as the DV was calculated as 52.7%. PD-partners with high MS score (beta = −.559,* p* ≤ .001) experienced less caregiver burden. A worsening of PD-patients' cognition increased the CBS score although it did not reach statistical significance (beta = .219, *p* = .050).

## 4. Discussion

### 4.1. Major Findings

Consistent with the result of other dyadic research with stroke patients and frail elderly, the average MS score was quite high and the patients tend to rate their mutuality higher than their caregivers [17, 18, 34]. However, in the present study the difference was not significant except for the dimension of reciprocity. This is the first study, to the best of our knowledge, to explore mutuality from the PD-patient's perspective. We found that PD-patients with high level of mutuality also experienced high HRQoL. Similar result has been shown in a study of patients with dementia [35]. Furthermore, having a male partner was associated with higher rated level of mutuality, but not with better HRQoL. Research so far is inconsistent regarding gender and HRQoL even if Martinez-Martin et al. (2008) observed more anxiety and worse HRQoL in female caregivers [4, 36]. Mutuality has also been reported as a protective factor of caregiver burden, which is in line with our result showing that PD-partners who perceived high mutuality also experience lower caregiver burden [9, 11, 37, 38].

Both motor and NMS were correlated with the MS, PDQ8SI, and CBS scores even though neither H/Y nor UPDRS III significantly contributed to the explained variances in the subsequent regression analysis. According to prior research, it seems that NMS such as depression, impaired cognition, sleep disorders, and fatigue have a larger impact on PD-patients' HRQoL than motor symptoms [3, 5, 39–41]. Similar result has also been reported regarding caregivers' mutuality showing gait impairment correlating with mutuality but not as a significant predictor [14]. The negative impact of NMS on PD-patients' HRQoL and impaired cognition on caregiver burden was expected and has also been reported in an extensive literature review by Chaudhuri [1].

Another interesting finding which has not been reported, as far as we know, was that mutuality scored by one member of the dyad was the strongest contributor of the level of mutuality experienced by the other member of the dyad. Using the modified stress-appraisal model but with a dyadic perspective [9, 20], some of our results could hypothetically be explained by the relatively high reported frequencies of NMS such as sleeping difficulties (55%), nocturia (78%), and restless legs (53%). These NMS, if protracted and severe enough, may not only disturb the PD-patients but also negatively affect their partners in various aspects such as sleep quality. Furthermore, impaired concentration (55%) and dizziness (65%), which were commonly reported in this study, may also affect the partners' wellbeing through worries of fall and need of adaptation or adjustment of daily activities. On the other hand, it may also affect the patients' wellbeing due to an increased experience of dependency and loss of the role as a partner. Altogether, the balance of responsibilities, interdependency, and roles may alter and put a strain on the relationship. This is reflected in our results by the significant difference in the MS dimension of reciprocity. However, if the dyads succeed to find gratification, meaning, and support, high mutuality may ameliorate negative outcomes such as burden and improve HRQoL even though the disease severity worsens by time. Overall, our results encourage a dyadic perspective due to the potential impact perceived mutuality has on HRQoL and caregiving burden when evaluating PD-symptoms and tailoring interventions. Paying attention to the experience of mutuality by both members of the dyad will allow clinicians to detect high risk dyads and look for interventions that address the patient's and their partner's wellbeing. Interventions such as social support, respite care, couple therapy, or counseling may help the couple to adapt and adjust to the ever changing care situation and find inner strength to cope. This will allow the medical system to provide a quality collaborative care that can improve patient outcomes and ameliorate caregiver burden.

### 4.2. Limitations

The present study has some limitations. First, we have only explored disease-related factors and mutuality association with the dependent variables of interest in our study. This is shown by the relatively low explained variance in the regression models. Future research would benefit from exploring models with measurement of both PD-related and general factors affecting the caregiving situation. However, our results provide a starting point for future studies with a dyadic perspective in PD. Secondly, the cross-sectional design and the rather small sample size with predominance of patients with mild to moderate PD limit the generalizability and possibility of causal inferences. Nevertheless, this was the initial analysis of baseline data within an ongoing longitudinal study, and we are anticipating data that will enable assessment of changes during the follow-up. Thirdly, the use of PDQ8 as measurement of HRQoL may not assess all suggested domains of the concept HRQoL. However, PDQ8 is a validated and a commonly used questionnaire in PD research and enabled us to compare our research with others.

## 5. Conclusion

The main findings of our study suggest that high level of mutuality experienced by the PD-patient was associated with their HRQoL. This was also shown in the PD-partner sample with an association between mutuality and burden. Furthermore, level of mutuality scored by one member of the dyad was shown to be a dominant contributor to the other member's mutuality. We do acknowledge that more research is needed including both PD-related and general factors in different PD settings. In general, it seems that NMS contribute to a larger extent to the mutual relationship in PD-affected dyads than motor disabilities.

## Figures and Tables

**Table 1:** Demographics and clinical features. *n* = 51 dyads.

		PD-patient	PD-partner
Female	*n* (%)	22 (43.1)	29 (56.9)
Level of education			
Elementary	*n* (%)	8 (15.7)	6 (11.8)
Secondary	*n* (%)	11 (21.6)	16 (31.4)
University	*n* (%)	32 (62.7)	29 (56.9)
Level of income (SEK)			
0–199,000	*n* (%)	13 (25.5)	13 (25.5)
200,000–450,000	*n* (%)	27 (52.9)	30 (58.8)
>450,000	*n* (%)	11 (21.6)	8 (15.7)
Retired^*∗*^	*n* (%)	45 (88.2)	39 (76.5)
Working	*n* (%)	10 (19.6)	16 (31.4)
Total MS	m (SD)	3.2 (0.65)	2.9 (0.77)
Dimension of MS			
Love	md (IQR)	3.6 (0.67)	3.6 (1.0)
Shared pleasurable activities	md (IQR)	3.2 (1.25)	3.0 (1.25)
Shared values	md (IQR)	3.0 (1.0)	3.0 (1.5)
Reciprocity	md (IQR)	3.3 (1.0)	2.8 (1.67)
Total CBS	m (SD)		42.5 (15.8)
PD-duration	m (SD)	8.4 (6.4)	
PDQ8SI	m (SD)	27.4 (14.6)	
IQCODE	M (SD)	3.2 (.53)	
Hohen & Yahr	md (IQR)	2.0 (1)	
NMSQuest	m (SD)	12.1 (4.6)	
UPDRS III	m (SD)	18.1 (5.8)	
PD-patients self-rating of motor signs			
Tremor	*n* (%)	28 (54.9)	
Bradykinesia	*n* (%)	43 (84.3)	
Rigidity	*n* (%)	38 (74.5)	
Gait	*n* (%)	35 (68.6)	

Notes: PD: Parkinson's disease, MS: mutuality scale, CBS: caregiver burden scale, PDQ8SI: the Parkinson's Disease Questionnaire Summery Index, IQCODE: Informant Questionnaire on Cognitive Decline in the Elderly, NMSQuest: Non-motor Symptoms Questionnaire, and UPDRS III: the Unified Parkinson's Disease Rating Scale-Part III.

^*∗*^Some of the study subjects were still working.

**Table 2:** PD-patients^*∗*^ self-rated frequency of non-motor symptoms. *n* = 51.

	Yes *n* (%)
Dribbling	22 (43.1)
*Taste/smelling*	*28 (54.9)*
Swallowing	19 (37.3)
Vomiting	7 (13.7)
Constipation	25 (49.0)
Bowel incontinence	8 (15.7)
*Bowel emptying incomplete*	*26 (51.0)*
*Urgency*	*38 (74.5)*
*Nocturia*	*40 (78.4)*
Pain	17 (33.3)
Weight	12 (23.5)
Remembering	25 (49.0)
Loss of interest	12 (23.5)
Hallucinations	15 (29.4)
*Concentrating*	*28 (54.9)*
Sad, blues	24 (47.1)
Anxiety	20 (39.2)
Sex drive	16 (31.4)
*Sex difficulty*	*27 (52.9)*
*Dizzy*	*33 (64.7)*
Falling	25 (49.0)
Day time sleepiness	11 (21.6)
*Insomnia*	*28 (54.9)*
Intense vivid dreams	16 (31.4)
Acting out during dreams	18 (35.3)
*Restless legs*	*27 (52.9)*
Swelling	12 (23.5)
Sweating	14 (27.5)
Diplopia	20 (39.2)
Delusions	6 (11.8)

^*∗*^PD: Parkinson's disease.

Italics: frequency > 50%.

**Table 3:** Frequency of PD-patients^*∗*^ who need help in different daily activities. *n* = 51.

	No help		Supervision		Some help		All help	
	*n*	(%)	*n*	(%)	*n*	(%)	*n*	(%)
Grooming/dressing	40	78.4	6	11.8	4	7.8	1	2.0
Bath/shower	38	74.5	6	11.8	5	9.8	2	3.9
Food intake	44	86.3	2	3.9	5	9.8	0	
Toileting	42	82.4	3	5.9	5	9.8	1	2.0
Walking/transferring	28	54.9	6	11.8	15	29.4	2	3.9
Cooking/cleaning	23	45.1	2	3.9	16	31.4	10	19.6
Shopping	19	37.3	1	2.0	21	41.2	10	19.6

^*∗*^PD: Parkinson's disease.

**Table 4:** Spearman rank correlation coefficients between independent variables and dependent variables. *n* = 51 dyads.

	PD-patient MS		PDQ8SI		PD-partner MS		CBS	
	Rho	*p* value	Rho	*p* value	Rho	*p* value	Rho	*p* value
PD-patient MS	1.00							
PDQ8SI	−.516	<.001	1.00					
PD-partner MS	.524	<.001	−.409	.003	1.00			
CBS	−.262	.063	.292	.038	−.631	<.001	1.00	
UPDRS III	−.311	.026	.322	.021	−.255	.071	.286	.042
H/Y	−.290	.039	.413	.003	−.309	.027	.336	.016
NMSQuest	−.178	.212	.631	<.001	−.252	.074	.258	.067
IQCODE	−.229	.107	.285	.042	−.529	<.001	.618	<.001
Cohabitation	.056	.695	−.013	.925	.072	.614	−.296	.035
PD-duration	−.199	.171	.085	.563	−.100	.495	.053	.715

Notes: PD: Parkinson's disease, PDQ8SI: the Parkinson's Disease Questionnaire Summery Index, MS: mutuality scale, CBS: caregiver burden scale, NMSQuest: the Non-motor Symptom Questionnaire, UPDRS III: the Unified Parkinson's Disease Rating Scale-Part III, H/Y: Hohen & Yahr, and IQCODE: Informant Questionnaire on Cognitive Decline in the Elderly.

**Table 5:** Multiple linear regression analysis to find predictors of the Parkinson's Disease Summary Index (PDQ8SI), caregiver burden scale, PD-patient mutuality, and PD-partner mutuality. *n* = 51 dyads.

	Unstandardized coefficients	Standardized coefficients	*p* value	95% CI	Tolerance/VIF
*Dependent variable = PD-patient MS* Adj *R*^2^ = .316					
Predictors					

*PD-partner MS*	.356	.419	*.002*	.143–.569	.882/1.134
UPDRS III	−.023	−.205	.113	−.052–.006	.852/1.174
NMS	−.025	−.176	.169	−.061–.011	.866/1.155
*PD-partner gender* ^**∗**^	.434	.332	*.017*	.080–.788	.759/1.318
Education^**∗***∗*^	.144	.110	.366	−.173–.461	.938/1.066
PD-partner age	−.001	−.016	.902	−.020–.018	.768/1.302

*Dependent variable = PDQ8SI* Adj *R*^2^ = .497					
Predictors					

*PD-patient MS*	−9.655	−.433	*.001*	−14.862–−4.449	.752/1.330
UPDRS III	.090	.036	.749	−.475–.655	.814/1.228
*NMS*	1.592	.498	*<.001*	.871–2.313	.803/1.245
IQCODE	.034	.032	.762	−.190–.257	.911/1.097
PD-partner gender^*∗*^	−4.329	−.148	.234	−11.565–2.907	.666/1.500
Education^*∗∗*^	−3.353	−.115	.275	−9.471–2.766	.924/1.083
PD-partner age	−.190	−.121	.293	−.551–.171	.778/1.285

*Dependent variable = PD-partner MS* Adj *R*^2^ = .289					
Predictors					

*PD-patient MS*	.542	.461	*.002*	.216–.869	.752/1.330
UPDRS III	−.013	−.101	.450	−.049–.022	.814/1.228
NMS	.011	.066	.621	−.034–.056	.803/1.245
*IQCODE*	−.017	−.314	*.016*	−.031–−.003	.911/1.097
PD-partner gender^*∗*^	.039	.025	.865	−.415–.492	.666/1.500
Education^*∗∗*^	−.117	−.076	.543	−.500–.267	.924/1.083
PD-partner age	−.009	−.104	.446	−.031–.014	.778/1.285

*Dependent variable = CBS* Adj *R*^2^ = .527					
Predictors					

*PD-partner MS*	−11.541	−.559	*<.001*	−16.149–−6.933	.771/1.296
UPDRS III	.447	.163	.129	−.136–1.030	.852/1.174
NMS	.282	.081	.449	−.462–1.026	.839/1.191
IQCODE	.251	.219	.050	.000–.503	.802/1.247
PD-partner gender^*∗*^	3.023	.095	.400	−4.143–10.188	.756/1.322
Education^*∗∗*^	1.178	.037	.713	−5.250–7.607	.931/1.074
PD-partner age	−.253	−.148	.191	−.636–.131	.768/1.302

Notes: PD: Parkinson's disease, PDQ8SI: the Parkinson's Disease Questionnaire Summery Index, MS: mutuality scale, CBS: caregiver burden scale, NMSQuest: the Non-motor Symptom Questionnaire, UPDRS III: the Unified Parkinson's Disease Rating Scale-Part III, and IQCODE: Informant Questionnaire on Cognitive Decline in the Elderly. Italics: significant predictors.

^*∗*^PD-partner gender = 0 = female, 1 = male.

^*∗∗*^Education = 0 = either elementary, secondary, or only one with university education and 1 = both with university education.
